# Association of BRCA Mutation Status with Clinical Outcomes in High-Grade Serous Ovarian Cancer

**DOI:** 10.3390/healthcare14091193

**Published:** 2026-04-29

**Authors:** Alexandru Marius Petrusan, Catalin Vladut Ionut Feier, Calin Muntean, Vasile Gaborean, Andrei Stefan Petrusan, Delia Nicoara, Emil Marius Puscas, Ioan Paul Tiberiu Puia, Andrei Pasca, Patriciu Achimaș-Cadariu

**Affiliations:** 1 Doctoral School, “Iuliu Hațieganu” University of Medicine and Pharmacy, 400012 Cluj-Napoca, Romania; petrusan.alexandru.marius@elearn.umfcluj.ro; 2 Department of Surgical Oncology and Gynecologic Oncology, “Iuliu Hațieganu” University of Medicine and Pharmacy, 400012 Cluj-Napoca, Romania; mariusemilpuscas@gmail.com (E.M.P.); pasca_andrei@elearn.umfcluj.ro (A.P.); pachimas@umfcluj.ro (P.A.-C.); 3 Department of Surgical Oncology, “Prof. Dr. I. Chiricuta” Institute of Oncology, 400015 Cluj-Napoca, Romania; medipuia@gmail.com; 4 Abdominal Surgery and Phlebology Research Center, “Victor Babeş” University of Medicine and Pharmacy Timişoara, 300041 Timisoara, Romania; 5 First Surgery Clinic, “Pius Brinzeu” Clinical Emergency Hospital, 300723 Timişoara, Romania; 6 Medical Informatics and Biostatistics, Department III-Functional Sciences, “Victor Babeş” University of Medicine and Pharmacy Timişoara, Eftimie Murgu Square No. 2, 300041 Timişoara, Romania; 7 Thoracic Surgery Research Center, “Victor Babeş” University of Medicine and Pharmacy Timişoara, 300041 Timişoara, Romania; vasile.gaborean@umft.ro; 8 Department of Surgical Semiology, “Victor Babeş” University of Medicine and Pharmacy Timişoara, 300041 Timişoara, Romania; 9 Faculty of Medicine, “Iuliu Hațieganu” University of Medicine and Pharmacy, 400012 Cluj-Napoca, Romania; petrusan.andrei.stefan@elearn.umfcluj.ro (A.S.P.); delianicoara13@gmail.com (D.N.); 10 Department of Quality Management, “Prof. Dr. I. Chiricuta” Institute of Oncology, 400015 Cluj-Napoca, Romania

**Keywords:** high-grade serous ovarian carcinoma, BRCA mutation, progression-free survival, cytoreductive surgery, residual disease, peritoneal carcinomatosis

## Abstract

**Background/Objectives:** High-grade serous ovarian carcinoma (HGSOC) is associated with high relapse rates despite aggressive multimodal treatment. BRCA mutations, present in a substantial subset of patients, confer homologous recombination deficiency and increased sensitivity to platinum-based chemotherapy. This study evaluated the association between BRCA mutation status and clinical outcomes, focusing on dissemination patterns, treatment allocation, perioperative parameters, and progression-free survival (PFS). **Methods:** This prospective single-center cohort included 133 consecutive patients with newly diagnosed HGSOC treated between January 2020 and December 2025. Primary treatment strategy (primary debulking surgery [PDS] or neoadjuvant chemotherapy [NACT]) was determined by multidisciplinary assessment. BRCA testing was performed using tumor tissue or germline analysis. Patients were followed for 24 months. PFS was analyzed using Kaplan–Meier estimates and Cox regression models. **Results:** Pathogenic BRCA mutations were identified in 39.1% of patients. BRCA-mutated tumors demonstrated significantly lower rates of peritoneal carcinomatosis (50% vs. 77.77%, *p* = 0.001) and were more frequently managed with PDS (59.6% vs. 41.8%, *p* = 0.048). Perioperative outcomes were comparable between groups. Disease progression occurred less frequently in BRCA-mutated patients (32.69% vs. 51.85%, *p* = 0.017). In univariate analysis, BRCA mutation was associated with a 48% reduction in progression risk (HR 0.52, 95% CI 0.27–0.99, *p* = 0.048). After adjustment for age, FIGO stage, and residual disease, BRCA mutation was not independently associated with progression (HR 0.57, *p* = 0.124), although a protective trend was observed, while residual disease remained a significant predictor. **Conclusions:** In this prospective cohort, BRCA mutation status was associated with distinct dissemination patterns and a significant reduction in progression risk in HGSOC. Although residual disease remained the strongest independent prognostic factor after multivariable adjustment, a trend toward improved PFS observed among BRCA-mutated patients supports the role of homologous recombination deficiency as a meaningful modifier of disease trajectory. These findings reinforce the clinical relevance of molecular stratification in the contemporary management of HGSOC.

## 1. Introduction

Ovarian cancer continues to represent one of the most lethal gynecologic malignancies worldwide, accounting for more than 300,000 newly diagnosed cases and over 200,000 deaths annually according to contemporary global cancer statistics [1]. Despite therapeutic progress, it remains the leading cause of death among cancers of the female reproductive tract, largely because no effective population-based screening strategy has been successfully implemented and the majority of patients are diagnosed at advanced stages of disease [1,2]. Epidemiologic data consistently demonstrate that approximately 70–75% of women present with FIGO stage III or IV tumors, typically characterized by diffuse transcoelomic peritoneal dissemination at the time of diagnosis [2,3]. As a consequence of late presentation and high recurrence rates, the five-year overall survival for patients with advanced-stage ovarian cancer remains below 40–45%, even in high-resource settings and despite improvements in surgical and systemic treatment strategies [1,4].

High-grade serous ovarian carcinoma (HGSOC) accounts for approximately 70% of epithelial ovarian cancers and represents the most aggressive histologic subtype [5,6]. It is characterized by marked genomic instability, near-universal TP53 mutations, and frequent defects in homologous recombination repair, including BRCA alterations [5,7]. Clinically, HGSOC commonly presents with extensive intraperitoneal dissemination and ascites. Although initial responses to platinum-based chemotherapy are frequent, most patients relapse within two to three years [5,8]. This pattern of early recurrence underscores the importance of molecular determinants of treatment response and prognosis.

Management of advanced HGSOC relies on the integration of maximal cytoreductive surgery and platinum-based chemotherapy [9,10]. The primary objective of upfront debulking surgery is complete macroscopic resection (R0), as residual disease volume remains the most significant prognostic factor influencing both progression-free and overall survival. A meta-analysis by Bristow et al. demonstrated a direct correlation between the extent of cytoreduction and survival, with incremental improvements observed as residual tumor burden decreased [9]. In patients with extensive peritoneal dissemination or low likelihood of optimal resection, neoadjuvant chemotherapy (NACT) followed by interval debulking surgery represents an evidence-based alternative [10,11]. Randomized trials such as EORTC 55971 and CHORUS have shown comparable overall survival between selected PDS and NACT strategies, with reduced perioperative morbidity in the NACT arm [11,12]. Therefore, treatment selection is guided by tumor burden, resectability assessment, and institutional surgical expertise.

Over the past decade, molecular stratification has substantially reshaped the management of HGSOC, particularly through the identification of BRCA mutations [5,13]. Pathogenic germline or somatic BRCA alterations are detected in approximately 20–40% of HGSOC cases and represent a major component of homologous recombination deficiency (HRD) [5,14]. BRCA proteins are critical for accurate repair of DNA double-strand breaks via homologous recombination; their loss leads to genomic instability, a defining feature of HGSOC [5]. Importantly, BRCA deficiency confers increased sensitivity to platinum-based chemotherapy, translating into improved response rates and prolonged progression-free survival [13,15].

The therapeutic impact of BRCA status has been further reinforced by the introduction of poly(ADP-ribose) polymerase (PARP) inhibitors, which exploit synthetic lethality by selectively targeting homologous recombination-deficient tumor cells [16,17,18]. Phase III trials have demonstrated a significant prolongation of progression-free survival with PARP inhibitor maintenance, particularly in BRCA-mutated patients, supporting the incorporation of BRCA testing into the initial evaluation of HGSOC [17,19]. Beyond its predictive value for targeted therapies, BRCA mutation status has also been associated with improved survival in observational cohorts; however, its prognostic effect appears to be closely interrelated with established clinical factors such as surgical outcome and treatment strategy [13].

In Romania, ovarian cancer continues to impose a considerable oncologic burden, with mortality rates exceeding those reported in several Western European countries, reflecting disparities in early detection and access to specialized care [20,21]. National data indicate that a substantial proportion of patients are diagnosed at advanced stages, often after referral delays or limited access to high-volume gynecologic oncology centers [20,22]. Historically, genetic counseling and BRCA testing were inconsistently available, restricting identification of hereditary cases [23]. Although access to molecular diagnostics and PARP inhibitors has expanded within tertiary oncology institutes in recent years, implementation remains uneven across regions [22,23]. Assessing BRCA mutation status in contemporary Romanian cohorts therefore provides meaningful real-world insight into the integration of molecular stratification into routine clinical practice.

In addition to survival outcomes, this study explores the association between BRCA mutation status and clinicopathological characteristics at diagnosis in a prospective real-world cohort, with particular attention to patterns of tumor dissemination.

Against this background, we undertook a comprehensive analysis to examine the relationship between BRCA mutation status and clinical outcomes in women with HGSOC, focusing specifically on tumor dissemination patterns, therapeutic management, perioperative parameters, and progression-free survival.

## 2. Materials and Methods

This single-center prospective observational cohort study with longitudinal follow-up was conducted at the “Prof. Dr. Ion Chiricuță” Institute of Oncology (IOCN), Cluj-Napoca, Romania. A total of 174 patients were initially assessed for eligibility. Of these, 21 patients opted to continue follow-up at other institutions, 12 were lost to follow-up, and 8 declined BRCA testing. The final study cohort included 133 patients.

### 2.1. Inclusion and Exclusion Criteria

#### 2.1.1. Inclusion Criteria

Patients were eligible if they met the following criteria:

1. Histologically confirmed high-grade serous ovarian carcinoma.
2. Completion of primary oncologic treatment (surgery and chemotherapy) within the institution.
3. Availability of tumor tissue and/or peripheral blood samples for BRCA mutation testing.
4. Signed informed consent for longitudinal follow-up.
5. Participation in scheduled postoperative surveillance visits for up to 24 months.

#### 2.1.2. Exclusion Criteria

Patients were excluded if they:

1. Had low-grade or non-epithelial ovarian malignancies.
2. Presented with recurrent disease at first evaluation.
3. Received initial oncologic treatment outside IOCN.
4. Had incomplete treatment or were lost to follow-up before outcome assessment.
5. Withdrew informed consent.

All enrolled patients underwent BRCA mutation analysis as part of the study protocol. Genetic testing was performed on tumor tissue specimens obtained at surgery or biopsy, while germline analysis was carried out using peripheral blood samples.

BRCA testing was performed using either tumor tissue or peripheral blood samples, depending on availability and clinical context at the time of diagnosis. Analyses were carried out in accredited laboratories using standard diagnostic protocols. In patients who underwent peripheral blood testing, detected BRCA mutations were considered germline. In contrast, in cases where testing was performed exclusively on tumor tissue, the origin of BRCA mutations (somatic versus germline) could not be systematically determined due to the lack of paired germline analysis. Therefore, BRCA mutation status was recorded as positive or negative irrespective of origin.

The study did not involve any experimental procedures or therapeutic interventions. The treatment was carried out in accordance with the institute’s standard protocols, as determined by the attending medical team.

### 2.2. Baseline Evaluation and Staging

At initial presentation, patients were systematically evaluated through clinical examination, including a detailed gynecologic assessment, together with serum CA-125 testing. Staging investigations comprised contrast-enhanced computed tomography of the thorax and abdomen, supplemented by pelvic magnetic resonance imaging to better define locoregional tumor extension and intraperitoneal disease involvement.

Tumor staging was determined in accordance with the International Federation of Gynecology and Obstetrics (FIGO 2014) [24] criteria. In addition to stage assignment, detailed pathological features were documented, including lymphatic and vascular invasion, involvement of adjacent structures, and the presence of peritoneal carcinomatosis.

### 2.3. Multidisciplinary Treatment Decision

Each case was reviewed in a multidisciplinary tumor board that included surgeons, medical oncologists, radiologists, and pathologists. Therapeutic strategy was determined by consensus after careful evaluation of imaging findings, the extent of peritoneal involvement, estimated surgical resectability, and the patient’s overall performance status, leading to the selection of either:

- Primary debulking surgery (PDS), or
- Neoadjuvant chemotherapy (NACT) followed by interval debulking surgery.

Surgical and Systemic Treatment

The objective of primary debulking surgery was to achieve complete macroscopic cytoreduction (R0). Residual disease was classified intraoperatively as either R0, defined by the absence of visible tumor, or R1, indicating the presence of macroscopic residual disease. Patients considered unsuitable for upfront optimal cytoreduction received platinum-based combination chemotherapy as neoadjuvant treatment, followed by interval debulking surgery when feasible. Operative time and perioperative parameters were systematically recorded, including duration of surgery, length of postoperative hospital stay, requirement for intensive care unit (ICU) admission, and duration of ICU stay. Patients received standard platinum-based chemotherapy. PARP inhibitors were administered in BRCA-mutated patients according to clinical indications

### 2.4. Follow-Up and Outcome Definition

Patients were prospectively monitored through structured follow-up visits conducted at predefined intervals over a maximum observation period of 24 months. Clinical surveillance was carried out in accordance with institutional protocols and included ongoing assessment for evidence of disease recurrence.

The primary endpoint of the study was progression-free survival (PFS), defined as the time interval between the date of initial surgical management and the first documentation of disease progression. Disease progression was established on the basis of radiologic findings indicative of recurrence, sustained elevation of serum CA-125 consistent with accepted criteria, or histopathologic confirmation when available.

The study protocol was reviewed and approved by the Ethics Committee of the “Ion Chiricuță” Oncology Institute, Cluj-Napoca (approval no. 166/15 December 2019). All procedures were conducted in accordance with the principles outlined in the Declaration of Helsinki and relevant national ethical regulations governing biomedical research.

### 2.5. Statistical Analysis

All statistical analyses were performed using IBM SPSS Statistics for Windows, Version 25.0 (IBM Corp., Armonk, NY, USA) and Jamovi, Version 2.26 (The Jamovi Project, Sydney, Australia). The distribution of continuous variables was assessed for normality using the Shapiro–Wilk test. Normally distributed variables were expressed as mean ± standard deviation (SD), while non-normally distributed variables were reported as median with interquartile range (IQR).

Categorical variables were summarized as frequencies and percentages. Comparisons between BRCA-mutated and BRCA wild-type groups were conducted using the independent samples Student’s *t*-test for parametric continuous variables and the Mann–Whitney U test for non-parametric variables. Associations between categorical variables were evaluated using the chi-square test or Fisher’s exact test when appropriate.

Cox proportional hazards regression models were used to estimate hazard ratios (HRs) with 95% confidence intervals (CIs). Univariate analyses were performed initially, followed by multivariate modeling including variables considered clinically relevant. The proportional hazards assumption was assessed by graphical inspection of survival curves.

All tests were two-sided, and a *p*-value < 0.05 was considered statistically significant.

## 3. Results

A total of 133 patients who underwent treatment for HGSOC at the “Prof. Dr. Ion Chiricuță” Institute of Oncology in Cluj-Napoca were included in this study between 1 January 2020 and 31 December 2025.

The mean age of the patients was 56.72 ± 9.36 years, with the majority originating from urban areas (62.4% vs. 37.6%).

BRCA mutation testing was performed using tissue-based analysis in 76 cases (57.1%) and germline testing in 57 cases (42.9%). A pathogenic BRCA mutation was identified in 52 patients (39.1%). The prevalence of BRCA mutations did not differ significantly between the two testing approaches (*p* = 0.469), with mutations detected in 29 patients (55.8%) through somatic testing and in 23 patients (44.2%) through germline analysis. Table 1 summarizes the demographic characteristics of the study population.

### 3.1. Key Information

The baseline clinicopathological characteristics of the study population are presented in Table 2.

Due to the low number of BRCA wild-type patients classified as FIGO stage I and II (<5 cases), an additional analysis was performed to assess potential differences in the distribution of advanced stages (FIGO III versus IV) according to BRCA mutation status. The results are presented in Table 3.

At the time of radiologic diagnosis, the mean length of the tumor’s longest axis was 9.49 ± 4.23 cm. A statistically significant difference was observed in mean tumor size between BRCA-mutated and BRCA wild-type patients (10.53 ± 4.74 cm vs. 8.83 ± 3.75 cm, respectively; *p* = 0.031). Patients with BRCA mutations demonstrated lower CA-125 levels, with a median of 473 [232 to 1505], compared with 697 [382 to 1684] in BRCA wild-type patients. This corresponds to an approximate 32% reduction in median CA-125 levels in the BRCA-mutated group. However, this difference did not reach statistical significance between the two groups (*p* = 0.309).

Prior to the initiation of either primary surgical treatment or neoadjuvant chemotherapy, peritoneal carcinomatosis was documented in 89 cases (66.91%). A significantly lower proportion of BRCA-mutated patients presented with peritoneal carcinomatosis compared to BRCA wild-type patients (26 cases (50%) vs. 63 cases (77.77%), *p* = 0.001).

Among BRCA-mutated patients, 31 (59.6%) underwent primary debulking surgery, while 21 (40.4%) received neoadjuvant chemotherapy. In contrast, a significantly lower proportion of wild-type patients underwent primary debulking surgery (33 patients, 41.8%; *p* = 0.048).

However, no statistically significant differences were observed between the two groups regarding the number of neoadjuvant chemotherapy cycles administered (7 ± 2.66 in the BRCA-mutated group vs. 6.42 ± 2.11 in the wild-type group; *p* = 0.695).

### 3.2. Perioperative and Postoperative Outcomes

The mean duration of surgical procedures was 104.90 ± 47.66 min, with no statistically significant difference observed between BRCA-mutated and BRCA wild-type patients (103.35 ± 48.33 min vs. 105.94 ± 47.55 min, respectively; *p* = 0.784).

With regard to postoperative outcomes, including length of hospital stay, the need for ICU monitoring, and duration of ICU stay, the results are summarized in Table 4.

### 3.3. Progression-Free Survival According to BRCA Status

No deaths were recorded among the patients during the follow-up period.

Disease progression was documented in 59 patients (44.36%). Among these cases, 9 patients had positive resection margins (R1), while 50 developed recurrence after complete cytoreduction.

Disease progression occurred significantly less frequently (*p* = 0.017) among BRCA-mutated patients (17 patients, 32.69%) compared to wild-type patients (42 patients, 51.85%). Consistently, BRCA-mutated tumors accounted for only 28.82% of all progression events.

Among the 18 patients with positive resection margins (R1), 9 (50%) developed disease progression. Of these, only one case (11.11%) occurred in a BRCA-mutated patient, while the remaining eight cases (88.89%) were observed in BRCA wild-type patients. The difference in proportions between the two groups was statistically significant (*p* < 0.001).

When focusing exclusively on patients who achieved complete cytoreduction (R0), recurrence occurred in 17 BRCA-mutated patients (34%) and in 33 BRCA wild-type patients (66%). A statistically significant difference was observed in the proportion of patients who subsequently developed recurrence according to BRCA mutation status (*p* = 0.032).

**Figure 1:**
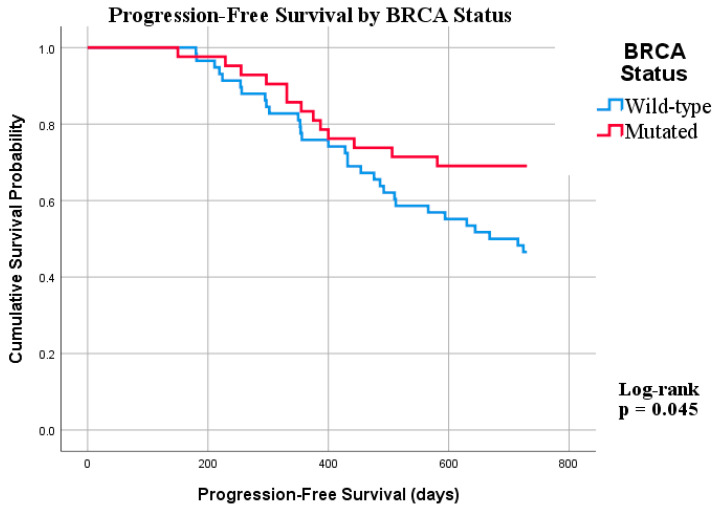
Progression-Free Survival by BRCA status.

To further evaluate the impact of BRCA mutation status on medium- and long-term prognosis, survival analyses were performed using the Kaplan–Meier method. PFS was estimated from the date of primary surgery to documented disease progression. At baseline, 81 BRCA wild-type and 52 BRCA-mutated patients were included in the analysis, with the number at risk progressively decreasing over the 730-day follow-up period. Survival curves were compared using the log-rank test. Results are presented in Figure 1 and Table 5.

The mean progression-free survival was 614.52 days (Standard Error (SE): 28.24) in the BRCA-mutated group and 563.88 days (SE: 25.38) in the BRCA wild-type group. The median PFS was not reached in the BRCA-mutated group due to an insufficient number of progression events during follow-up, while in the BRCA wild-type group the estimated median PFS was 668 days.

A total of 59 progression events occurred during 1950.5 person-months of follow-up, corresponding to an incidence rate of 3.02 events per 100 person-months.

The Kaplan–Meier curves demonstrate a clear separation between the two groups, with BRCA-mutated patients showing a consistently higher progression-free survival probability throughout the follow-up period. This difference becomes apparent early and is maintained over time, supporting a sustained reduction in progression risk in the BRCA-mutated group.

In addition to this, to evaluate the impact of BRCA mutation status on progression-free survival, a Cox proportional hazards regression analysis was performed. The overall model was statistically significant (*p* = 0.031). In univariate analysis, BRCA-mutated patients demonstrated a significantly reduced risk of disease progression compared to wild-type patients (HR 0.52, 95% CI 0.27–0.99, *p* = 0.048). Specifically, the presence of a BRCA mutation was associated with an approximately 48% reduction in the hazard of progression during the follow-up period. The results are presented in Table 6.

Given the significant baseline differences observed between BRCA-mutated and wild-type patients in terms of age and FIGO stage, a multivariate Cox proportional hazards model was constructed to adjust for these potential confounders, together with residual disease status. The overall model was statistically significant (*p* = 0.016). After adjustment, BRCA mutation status was no longer independently associated with progression-free survival (HR 0.57, 95% CI 0.27–1.168, *p* = 0.124), although a protective trend persisted. Age (HR 0.99, CI 0.96–1.03, *p* = 0.817) and FIGO stage (HR 1.37, CI 0.86–2.16, *p* = 0.184) were not significant predictors of progression. In contrast, residual disease remained an independent prognostic factor, conferring a 2.5-fold increased risk of progression (HR 2.54, CI 1.19–5.43, *p* = 0.016). The results are presented in Table 7.

## 4. Discussion

High-grade serous ovarian carcinoma continues to be the most lethal malignancy of the female reproductive tract, largely because it is commonly diagnosed at an advanced stage and is characterized by a high rate of relapse despite aggressive multimodal treatment [4,6]. Within our prospective cohort of 133 patients managed between 2020 and 2025, pathogenic BRCA mutation was identified in 39.1% of cases. BRCA-mutated tumors were associated with differences in peritoneal dissemination patterns and were more frequently managed with primary cytoreduction, likely reflecting variations in disease distribution at presentation. These features were accompanied by a longer progression-free survival compared with BRCA wild-type disease. Beyond previously reported survival differences, our findings provide additional insight into variations in tumor dissemination patterns associated with BRCA status in a real-world clinical setting, which may contribute to differences in disease behavior and initial presentation.

When adjusted for FIGO stage and residual disease, BRCA mutation status was no longer independently associated with progression, underscoring the dominant prognostic role of surgical radicality in advanced HGSOC. These findings are consistent with the well-established principle that the extent of cytoreduction remains the most critical determinant of patient outcome, outweighing molecular characteristics in multivariable models. In this context, the observed reduction in progression risk among BRCA-mutated patients should be interpreted within the broader framework of tumor burden and surgical outcome. Nonetheless, the consistent trend toward improved progression-free survival in BRCA-mutated patients supports the notion that homologous recombination deficiency may contribute to tumor biology and therapeutic responsiveness in HGSOC, acting in conjunction with, rather than independently of, key clinical factors such as residual disease [5,7,9].

The prevalence of pathogenic BRCA variants in our cohort (39.1%) falls within the range reported in large-scale genomic studies of HGSOC. Comprehensive molecular characterization by The Cancer Genome Atlas (TCGA) identified homologous recombination pathway alterations in approximately half of HGSOC cases, with BRCA mutations accounting for a significant proportion of these defects [5]. In a complementary analysis, Pennington et al. reported deleterious alterations in homologous recombination genes in nearly one-third of ovarian carcinomas, further supporting the substantial contribution of BRCA-associated genomic instability in this disease [14]. The concordance between our findings and these landmark datasets suggests that the molecular profile of the present cohort is consistent with broader genomic observations in HGSOC.

BRCA-mutated patients in our cohort were significantly younger than BRCA wild-type patients (53.88 ± 9.18 vs. 58.54 ± 9.07 years, *p* = 0.005). This age difference is consistent with prior large cohort analyses demonstrating earlier disease onset among BRCA mutation carriers compared with non-carriers [13,25]. Nevertheless, in line with previous multivariable survival models, chronological age did not independently predict progression risk once stage and residual disease were taken into account, suggesting that tumor biology and treatment-related factors exert a stronger influence on outcome than age alone [15,26].

### 4.1. Tumor Dissemination Patterns and Biological Implications

Although the overall distribution of FIGO stages differed significantly between groups (*p* = 0.003), this difference was no longer evident when the analysis was restricted to advanced disease (FIGO III–IV), where stage distribution was comparable (*p* = 0.386). These findings are consistent with population-based studies indicating that BRCA mutation carriers do not systematically present with more advanced-stage disease compared with non-carriers [27,28]. Taken together, the data suggest that BRCA status does not substantially influence stage at diagnosis once invasive HGSOC has developed, and that differences in outcome are more likely driven by biological behavior and treatment response rather than initial stage distribution [29,30,31].

In contrast, peritoneal carcinomatosis was observed significantly less frequently among BRCA-mutated patients (50% vs. 77.77%, *p* = 0.001). Extensive transcoelomic dissemination represents a defining feature of advanced HGSOC and substantially increases surgical complexity as well as the likelihood of residual disease [26]. The 27% absolute reduction in carcinomatosis within the BRCA-mutated subgroup may reflect differences in tumor dissemination biology rather than stage distribution alone. Homologous recombination deficiency (HRD), a hallmark of BRCA-associated tumors, is characterized by genomic instability that may influence clonal evolution and metastatic potential, thereby contributing to variations in intraperitoneal spread [7,32].

Interestingly, BRCA-mutated tumors exhibited a greater mean maximal diameter at diagnosis (10.53 ± 4.74 cm vs. 8.83 ± 3.75 cm, *p* = 0.031), despite the lower prevalence of peritoneal carcinomatosis observed in this subgroup. This apparent discrepancy suggests that primary tumor bulk and metastatic dissemination may represent partially independent biological dimensions of HGSOC progression. As previously highlighted, ovarian cancer-related mortality is driven predominantly by diffuse transcoelomic spread rather than by the size of the primary adnexal mass [33,34]. Our findings align with this paradigm, indicating that larger tumor size does not necessarily translate into a more extensive peritoneal disease burden.

Baseline CA-125 levels were numerically lower in BRCA-mutated patients, although the difference did not reach statistical significance (*p* = 0.309). Given the documented association between CA-125 concentration and peritoneal tumor load, this trend may reflect reduced intraperitoneal dissemination rather than intrinsic differences in biomarker production [35,36].

### 4.2. Surgical Strategy and Perioperative Outcomes

BRCA-mutated patients were more frequently managed with primary debulking surgery (59.6% vs. 41.8%, *p* = 0.048), a finding likely related to the lower burden of peritoneal dissemination observed in this subgroup and the consequent higher probability of achieving optimal cytoreduction. The prognostic relevance of complete macroscopic resection has been consistently demonstrated, with residual disease recognized as the most powerful determinant of survival in advanced HGSOC [26,37].

Notably, although surgical allocation differed between groups, operative time and early postoperative parameters were comparable. Duration of surgery (*p* = 0.784), length of hospitalization (7.40 vs. 7.77 days, *p* = 0.784), rates of ICU admission (approximately 51% in both groups, *p* = 0.890), and ICU stay duration (*p* = 0.858) did not differ significantly. These data suggest that BRCA status does not appear to influence perioperative morbidity or short-term recovery. The absence of differences in immediate postoperative outcomes supports the validity of the observed survival contrasts, indicating that PFS differences are unlikely to be confounded by disparities in surgical tolerance or postoperative course [38,39,40].

Although the difference in residual disease rates between BRCA-mutated and wild-type patients did not reach statistical significance (7.69% vs. 17.28%), the observed trend may still be of clinical interest and is broadly consistent with prior evidence suggesting more favorable surgical outcomes among BRCA carriers. Several studies have reported that BRCA-associated tumors may exhibit increased chemosensitivity and potentially more favorable cytoreductive profiles, which could facilitate higher rates of complete macroscopic resection [25,41]. Given the well-established prognostic impact of residual disease in advanced HGSOC, even relatively small differences in cytoreduction rates may influence clinical outcomes, although this relationship should be interpreted with caution in the absence of statistical significance [26,37,39].

### 4.3. Progression-Free Survival

An important finding of the present analysis relates to progression-free survival. Disease progression was observed in 32.69% of BRCA-mutated patients compared with 51.85% of BRCA wild-type patients (*p* = 0.017). Notably, although BRCA-mutated cases accounted for 39.1% of the cohort, they represented only 28.82% of all progression events, underscoring a disproportionate reduction in recurrence burden.

In univariate Cox analysis, BRCA mutation was associated with a 48% reduction in the hazard of progression (HR 0.52, 95% CI 0.27–0.99, *p* = 0.048), a magnitude closely aligned with previously reported pooled hazard ratios demonstrating improved outcomes among BRCA1/2 carriers [13,15]. Importantly, within the R0 subgroup, recurrence remained significantly less frequent in BRCA-mutated patients (34% vs. 66%, *p* = 0.032), indicating that the protective effect of BRCA status persists even after complete macroscopic cytoreduction. This observation suggests that factors beyond surgical radicality may also contribute to the observed differences in progression risk.

From a biological perspective, these findings are mechanistically plausible. BRCA deficiency impairs the homologous recombination repair pathway, thereby reducing the cell’s ability to accurately repair platinum-induced DNA double-strand breaks [16,42]. This mechanism is generally associated with increased platinum sensitivity, which may contribute to deeper initial responses and delayed recurrence. The clinical relevance of this vulnerability has been further supported in the maintenance setting, where PARP inhibition significantly prolongs progression-free survival in BRCA-mutated HGSOC, reinforcing the concept that homologous recombination deficiency is linked to enhanced therapeutic sensitivity [17,43,44,45].

In multivariate analysis adjusting for age, FIGO stage, and residual disease, BRCA mutation did not retain independent statistical significance (HR 0.57, *p* = 0.124), whereas residual disease remained a strong independent predictor of progression (HR 2.54, *p* = 0.016). This attenuation suggests that the observed effect of BRCA status may be partially mediated through its association with tumor burden and resectability. Nonetheless, the persistent protective trend and the significant difference observed within the R0 subgroup indicate that BRCA mutation may still contribute to disease behavior and treatment response, although within a broader clinical context dominated by established prognostic factors.

While our findings demonstrate a reduced risk of progression and distinct clinical characteristics in BRCA-mutated patients, these results should be interpreted in the context of heterogeneous evidence reported in the literature. Although multiple studies have described improved survival outcomes among BRCA mutation carriers, the independent prognostic role of BRCA status remains debated, particularly after adjustment for established clinical factors such as stage and residual disease [17,19,41,46,47]. In several analyses, the survival advantage associated with BRCA mutations was attenuated in multivariate models, suggesting that its effect may be partially mediated through treatment-related variables rather than intrinsic tumor biology alone [41,46]. Moreover, improved progression-free survival in BRCA-mutated patients has been strongly linked to enhanced platinum sensitivity and the use of maintenance PARP inhibitors, which may act as major confounding factors in contemporary cohorts [17,19]. Therefore, the protective trend observed in our study, despite the lack of independent statistical significance in multivariate analysis, may reflect a combination of biological and therapeutic influences. Additionally, variations in treatment allocation, surgical outcomes, and real-world clinical practices may further contribute to differences observed across studies.

The interpretation of progression-free survival in the present cohort should also be considered within the context of disease-specific clinical dynamics. In high-grade serous ovarian cancer, the majority of recurrences occur within the first two to three years following initial treatment, making early progression a clinically relevant indicator of disease behavior [6,8,15]. In this setting, the 24-month follow-up captures a substantial proportion of progression events and provides meaningful insight into early treatment response and tumor biology [6,13,15]. At the same time, it is well recognized that progression-free survival does not necessarily translate into overall survival benefit, particularly in the context of effective post-progression therapies such as PARP inhibitors [17,19,43,44,45]. Consequently, the observed differences in progression risk should be interpreted as reflecting intermediate-term disease control rather than definitive long-term prognostic impact [17,19,47].

An important consideration in the interpretation of our findings relates to the potential biological heterogeneity within the BRCA-mutated cohort. In the present study, BRCA status was assessed irrespective of mutation origin, and a systematic distinction between germline and somatic alterations was not feasible in all cases. This distinction is not merely technical but biologically meaningful, as demonstrated by large genomic studies highlighting the complexity of homologous recombination deficiency in ovarian cancer [5,14]. Germline BRCA mutations confer a constitutive homologous recombination deficiency present across all tumor cells, reflecting a stable and systemic defect in DNA repair mechanisms [5,15]. In contrast, somatic alterations may arise within specific tumor clones and exist within a more heterogeneous genomic landscape, as shown in whole-genome analyses of ovarian cancer evolution [7]. As a result, these entities may differ in terms of clonal stability, evolutionary dynamics, and potentially therapeutic sensitivity, consistent with the concept of “BRCAness” and tumor heterogeneity described in prior studies [7,31].

By analyzing germline and somatic mutations as a unified category, the present study may have introduced a degree of biological heterogeneity that could attenuate the strength of observed associations, particularly in survival analyses, as suggested by prior reports evaluating BRCA-related outcomes [13,47]. This may partially explain the loss of statistical significance in multivariable models despite a consistent protective trend observed in univariate analysis [13,15]. Therefore, the impact of BRCA status on clinical outcomes in this cohort should be interpreted with caution, acknowledging that differential effects according to mutation origin could not be fully explored.

Although the association between BRCA mutation status and improved progression-free survival has been repeatedly described, the contribution of the present study lies in a different domain. Previous reports have predominantly focused on survival endpoints, imaging correlates, or selected treatment populations, and studies addressing dissemination patterns and surgical outcomes have mainly been retrospective or restricted to specific clinical settings. In contrast, our prospective real-world cohort allowed the joint evaluation of BRCA status in relation to disease distribution at diagnosis, multidisciplinary treatment allocation, perioperative course, and early progression-free survival within a contemporary care pathway. In this context, the most clinically informative aspect of our findings is that BRCA-mutated tumors were less frequently associated with peritoneal carcinomatosis and were more often selected for primary debulking surgery, while short-term perioperative outcomes were comparable between groups. These observations suggest that the relevance of BRCA status in HGSOC may extend beyond treatment sensitivity alone and may also be associated with differences in initial disease presentation and therapeutic management in routine clinical practice.

### 4.4. Study Limitations

Several limitations warrant consideration. BRCA testing was not fully standardized across the cohort, as patients underwent either tumor-based or germline analysis depending on resource availability, without systematic complementary confirmation. This approach reflects real-world clinical practice during the study period but may have introduced a potential risk of misclassification, particularly regarding the distinction between somatic and germline mutations. However, further stratification according to mutation origin was not feasible given the sample size, as this would have resulted in small subgroups with limited statistical power and potentially unreliable estimates. FIGO staging was assigned by different clinicians, introducing potential variability in staging assessment, and surgical procedures were performed by multiple surgeons, which may have influenced operative complexity. The single-center design and moderate sample size limit external generalizability and also constrain the number of variables that could be included in the multivariate model in order to avoid overfitting. Finally, as patient enrollment spanned the COVID-19 pandemic period, variations in referral patterns, surgical scheduling, and access to systemic therapies may have subtly influenced treatment allocation and progression dynamics.

## 5. Conclusions

In this prospective cohort of patients with HGSOC, BRCA mutation status was associated with distinct patterns of disease dissemination, a lower prevalence of peritoneal carcinomatosis, and differences in initial treatment allocation, alongside a reduced risk of progression. While residual disease remained the strongest independent predictor of progression, the consistent trend toward improved PFS observed among BRCA-mutated patients supports the role of homologous recombination deficiency as a modifier of disease trajectory rather than an independent prognostic determinant.

Beyond its established predictive value for targeted therapies, BRCA status may also contribute to variations in tumor presentation and therapeutic decision-making in routine clinical practice. These findings highlight the importance of integrating molecular and surgical factors in the management of HGSOC and support a more nuanced understanding of BRCA status within the broader clinical context.

## Figures and Tables

**Table 1:** Demographic characteristics.

Variables	All (n = 133)	BRCA Mutated (n = 52)	BRCA Wild-Type (n = 81)	*p*
Age (years, M ± SD)	56.72 ± 9.36	53.88 ± 9.18	58.54 ± 9.07	0.005
Environment				
Urban	83 (62.4%)	37 (71.2%)	46 (56.8%)	0.095
Rural	50 (37.6%)	15 (28.8%)	35 (43.2%)	

M = mean; SD = Standard Deviation.

**Table 2:** Baseline Tumor Characteristics Stratified by BRCA Status.

Variables	All (n = 133)	BRCA Mutated (n = 52)	BRCA Wild-Type (n = 81)	*p*
FIGO stage				0.003
I	10 (7.51%)	7 (13.46%)	3 (3.7%)	
II	8 (6.01%)	7 (13.46%)	1 (1.23%)	
III	84 (63.15%)	30 (57.69%)	54 (66.66%)	
IV	31 (23.3%)	9 (17.3%)	22 (27.16%)	
Lymphatic invasion	56 (42.1%)	20 (38.46%)	36 (44.44%)	0.385
Vascular invasion	15 (11.27%)	5 (9.61%)	10 (12.19%)	0.582
Adjacent organ invasion	41 (30.82%)	16 (30.76%)	25 (30.48%)	0.981
Residual tumor (R1)	18 (13.53%)	4 (7.69%)	14 (17.28%)	0.104

**Table 3:** Distribution of FIGO Stage III and IV According to BRCA Status.

Variables	All (n = 115)	BRCA Mutated (n = 39)	BRCA Wild-Type (n = 76)	*p*
FIGO stage				0.386
III	84 (63.15%)	30 (57.69%)	54 (66.66%)	
IV	31 (23.3%)	9 (17.3%)	22 (27.16%)	

**Table 4:** Postoperative outcomes.

Variables	All (n = 133)	BRCA Mutated (n = 52)	BRCA Wild-Type (n = 81)	*p*
Postoperative hospital stay (days, M ± SD)	7.63 ± 3.80	7.40 ± 1.65	7.77 ± 3.69	0.784
Postoperative ICU stay YES	68 (51.12%)	27 (51.92%)	41 (50.61%)	0.890
Postoperative ICU stay (days, M ± SD)	2.65 ± 1.7	2.61 ± 1.43	2.70 ± 1.48	0.858

M = mean; SD = Standard Deviation.

**Table 5:** Number at risk.

Time (Days)	0	200	400	600	730
Wild-type	81	79	60	45	39
Mutated	52	51	40	36	35

**Table 6:** Cox regression-BRCA mutated patients.

Variables in the Equation								
	B	SE	Wald	df	Sig.	Exp(B)	95.0% CI for Exp(B)	
							Lower	Upper
BRCA Mutated	−0.650	0.331	3.860	1	0.048	0.522	0.273	0.998

**Table 7:** Multivariate Cox regression for disease progression.

Variables in the Equation								
	B	SE	Wald	df	Sig.	Exp(B)	95.0% CI for Exp(B)	
							Lower	Upper
BRCA MUTATED	−0.563	0.367	2.360	1	0.124	0.569	0.277	1.168
AGE (years)	−0.004	0.018	0.054	1	0.817	0.996	0.962	1.031
FIGO stage	0.311	0.234	1.768	1	0.184	1.365	0.863	2.161
R1	0.934	0.387	5.829	1	0.016	2.545	1.192	5.432

## Data Availability

Data available on request due to patient confidentiality, privacy protection regulations (including GDPR), and institutional policies governing access to clinical data.
